# Engineered lung cell targeting and SLC7A11 siRNA expressing bacterial extracellular vesicles impair the progression of none‐small cell lung cancer

**DOI:** 10.1002/btm2.70021

**Published:** 2025-04-16

**Authors:** Xiao‐dan Wan, Xue‐liang Zhou, Jin‐long Liu, Hua Xu

**Affiliations:** ^1^ Department of Electrocardiogram, The First Affiliated Hospital Nanchang University Nanchang Jiangxi China; ^2^ Department of Cardiac Surgery, The First Affiliated Hospital Nanchang University Nanchang Jiangxi China; ^3^ Institute of Translational Medicine Shanghai University Shanghai China; ^4^ Department of Thoracic Surgery, The First Affiliated Hospital Nanchang University Nanchang Jiangxi China

**Keywords:** engineered bacterial extracellular vesicles, ferroptosis, none‐small cell lung cancer, SLC7A11

## Abstract

Non‐small cell lung cancer (NSCLC) presents significant therapeutic challenges, often characterized by aggressive proliferation and metastasis. This study investigates the role of SLC7A11, a ferroptosis‐related gene, in NSCLC progression and the potential of engineered bacterial extracellular vesicles (BEVs) expressing SLC7A11‐targeting siRNA as a therapeutic strategy. Using TCGA and GEO databases, we identified that SLC7A11 was significantly upregulated in NSCLC tissues. Functional assays demonstrated that SLC7A11 knockdown in NSCLC cell lines (NCI‐H2122 and NCI‐H647) via qPCR, Western blot, and immunofluorescence resulted in impaired proliferation, migration, and invasion abilities. In vivo xenograft models further revealed that SLC7A11 knockdown inhibited tumor growth and metastasis, corroborated by histological analyses. To enhance targeted delivery of SLC7A11 siRNA, we engineered BEVs with a lung cell targeting peptide, verifying their structure and function through transmission electron microscopy (TEM) and nanoparticle tracking analysis (NTA). In vivo toxicity assessments indicated safety for these bioengineered vesicles. Importantly, treatment with BEVs‐LCTP‐siSLC7A11 not only impaired tumorigenesis but also activated ferroptosis pathways, as evidenced by altered expression levels of SLC7A11 and transferrin in tumor and metastatic tissues. Our findings suggest that targeting SLC7A11 through engineered BEVs presents a promising approach to inhibit NSCLC progression while activating ferroptosis, offering insights into novel therapeutic strategies against lung cancer.

**Translational Impact Statement**

This study demonstrates that targeting SLC7A11—a ferroptosis‐linked gene overexpressed in NSCLC—using lung‐targeted bacterial extracellular vesicles (BEVs) delivering shRNA inhibits tumor growth, metastasis, and activates ferroptosis. Engineered BEVs enable safe, precise delivery, combining gene silencing and iron‐dependent cell death to combat aggressive NSCLC, offering a novel therapeutic strategy with clinical potential.

## 1 INTRODUCTION

Lung cancer remains one of the leading causes of cancer‐related mortality worldwide, with non‐small cell lung cancer (NSCLC) accounting for approximately 85% of all lung cancer cases.^1^, ^2^, ^3^, ^4^, ^5^ NSCLC is characterized by its heterogeneous nature and varied responses to existing treatment modalities, including surgery, chemotherapy, and targeted therapies.^6^ Despite advancements in therapeutic strategies, the prognosis for patients diagnosed with advanced‐stage NSCLC remains poor, underscoring the pressing need for innovative approaches to manage this malignancy.^7^, ^8^, ^9^

Ferroptosis is a form of regulated cell death distinct from apoptosis, necrosis, and autophagy, characterized by iron‐dependent lipid peroxidation leading to cellular damage.^10^, ^11^ Emerging evidence suggests that ferroptosis plays a crucial role in tumor biology, particularly in the context of cancer therapy resistance.^12^, ^13^ Unlike traditional forms of cell death, ferroptosis can circumvent mechanisms of drug resistance, making it an attractive target for novel therapeutic strategies.^14^, ^15^ Recent studies have shown that various cancer cells exhibit altered iron metabolism, contributing to their susceptibility or resistance to ferroptosis stimuli.^16^, ^17^ Notably, the cystine/glutamate antiporter system Xc‐, which is encoded by the SLC7A11 gene, is pivotal in maintaining intracellular redox homeostasis through the uptake of cysteine, a precursor for glutathione synthesis.^18^, ^19^ Dysregulation of SLC7A11 has been implicated in promoting tumorigenesis and metastasis in several cancers, including NSCLC. Therefore, targeting SLC7A11 represents a promising strategy to induce ferroptosis and inhibit the progression of NSCLC.^20^, ^21^

SLC7A11 is part of the solute carrier family of transporters and is primarily responsible for the uptake of cysteine into cells.^22^ In the tumor microenvironment, where oxidative stress and nutrient deprivation are prevalent, upregulation of SLC7A11 facilitates increased cysteine uptake, thereby enhancing glutathione levels and providing a survival advantage against oxidative damage.^23^ This mechanism has been associated with aggressive behaviors in cancer, including increased proliferation, migration, and invasion.^24^ In NSCLC, elevated expression of SLC7A11 has been linked to poor patient outcomes, suggesting its potential as a biomarker for disease progression.^25^ Furthermore, inhibiting SLC7A11 not only reduces cellular antioxidant defenses but also sensitizes cancer cells to ferroptosis.^26^ Thus, employing strategies to downregulate SLC7A11 could potentiate ferroptosis as a viable therapeutic avenue for NSCLC treatment. Despite the promise of targeting SLC7A11, several challenges remain, particularly in terms of effective delivery systems to ensure selective targeting of cancer cells while minimizing off‐target effects.^27^ Traditional drug delivery methods often face limitations such as low bioavailability, rapid clearance, and systemic toxicity. To enhance the specificity and efficacy of SLC7A11‐targeting therapies, advanced delivery systems are required.

Bacterial extracellular vesicles (BEVs) have emerged as versatile nanocarriers for RNA‐based therapeutics due to their natural ability to encapsulate and deliver biologically active molecules.^28^ BEVs possess intrinsic properties that facilitate cellular uptake, enabling them to traverse biological barriers and target specific tissues.^29^, ^30^ Importantly, engineering BEVs to carry small interfering RNA (siRNA) targeting SLC7A11 offers a promising strategy to inhibit its expression selectively within NSCLC cells.^31^, ^32^, ^33^ The engineering of BEVs involves the incorporation of specific peptides or ligands that enhance their affinity for target cells. In this study, we have designed BEVs to express a lung cell‐targeting peptide (LCTP) alongside SLC7A11‐targeting siRNA. The fusion of LCTP with the BEVs provides a mechanism for preferentially delivering therapeutic agents to lung cells, thereby improving treatment efficacy while reducing systemic exposure and potential side effects. The versatility of BEVs in accommodating various payloads, combined with their biocompatibility, positions them as an attractive platform for cancer therapeutics. Moreover, utilizing bacterial systems for BEV production allows for scalable and cost‐effective manufacturing processes, paving the way for future clinical applications.

This research aims to elucidate the role of SLC7A11 in NSCLC biology and explore the potential of engineered BEVs as a novel strategy for targeted therapy. By focusing on inducing ferroptosis through the downregulation of SLC7A11, we aim to overcome traditional treatment resistance mechanisms prevalent in NSCLC. Ultimately, this study could pave the way for innovative therapeutic approaches that harness the unique properties of BEVs, presenting a multifaceted strategy to combat the challenges associated with NSCLC treatment.

## 2 MATERIALS AND METHODS

### 2.1 Cell lines and culture

Human NSCLC cell lines, NCI‐H2122 and NCI‐H647, were obtained from the American Type Culture Collection (ATCC). Cells were cultured in RPMI‐1640 medium supplemented with 10% fetal bovine serum and 1% penicillin–streptomycin at 37°C in a humidified atmosphere containing 5% CO_2_. The culture medium was changed every 2–3 days to maintain optimal growth conditions.

### 2.2 Patient samples and data analysis

The expression levels of ferroptosis‐related genes were analyzed using publicly available datasets from the Cancer Genome Atlas (TCGA) and the Gene Expression Omnibus (GEO, GSE33532). Data retrieval and normalization were performed using R programming software with appropriate Bioconductor packages. Genes of interest, particularly SLC7A11, were evaluated for differential expression between tumor tissues and adjacent normal tissues.

### 2.3 siRNA design and transfection

siRNA targeting SLC7A11 was designed and synthesized by Genelily BioTech Co., Ltd. (Shanghai, China). The sequences used were: SLC7A11 siRNA: 5′‐GGAACUUGUCUUUCAGAAUTT‐3′; Control non‐targeting control (si‐NC): provided by Genelily BioTech Co., Ltd. (Shanghai, China). Transfection was performed using Lipofectamine RNAiMAX reagent (Thermo Fisher Scientific) according to the manufacturer's instructions. Briefly, cells were seeded in 6‐well plates at a density of 1 × 10^5^ cells per well and allowed to adhere overnight. The siRNA was then mixed with the transfection reagent and added to the cells. After 48 h, cells were collected for further analysis.

### 2.4 Quantitative polymerase chain reaction (qPCR)

Total RNA was isolated from cultured NSCLC cells or snap‐frozen tumor tissues using TRIzol reagent (Invitrogen, USA) following the manufacturer's protocol. Briefly, cells/tissues were homogenized in TRIzol (1 mL per 100 mg tissue), followed by chloroform phase separation. RNA was precipitated with isopropanol, washed with 75% ethanol, and dissolved in nuclease‐free water. RNA concentration and purity were verified using a NanoDrop 2000 spectrophotometer (Thermo Fisher Scientific, USA), with acceptable A260/A280 ratios of 1.8–2.1 and A260/A230>1.7. RNA integrity was confirmed by 1% agarose gel electrophoresis, displaying intact 28S/18S ribosomal RNA bands. For cDNA synthesis, 1 μg of total RNA was reverse‐transcribed using the PrimeScript RT Reagent Kit (Takara, Japan) in a 20 μL reaction volume under the following conditions: 37°C for 15 min (reverse transcription) and 85°C for 5 s (enzyme inactivation). qPCR amplification was performed in triplicate using SYBR Green PCR Master Mix (Applied Biosystems, USA) on a CFX96 Real‐Time PCR System (BioRad, USA). Each 20 μL reaction contained 2 μL cDNA template, 10 μL SYBR Green mix, 0.8 μL each primer (10 μM), and 6.4 μL nuclease‐free water. Thermocycling parameters: 95°C for 10 min (initial denaturation), followed by 40 cycles of 95°C for 15 s and 60°C for 1 min. Melt curve analysis (60–95°C, 0.3°C/s increment) confirmed primer specificity. No‐template and no‐reverse‐transcriptase (no‐RT) controls were included to rule out genomic DNA contamination. Gene expression was normalized to *GAPDH* using the 2^(−ΔΔ*Ct*)^ method.

The following primers were used: SLC7A11 Forward: 5′‐CCTGGACACTTTGTGCTGAC‐3′; SLC7A11 Reverse: 5′‐GCCACTGATGGTGTTGATCC‐3′; Transferrin Forward: 5′‐GCTGTCCCTGACAAAACGGT‐3′; Transferrin Reverse: 5′‐GTCACGGAAGCTGATGCACT‐3′; GAPDH Forward: 5′‐GTCAGTGGTGGACCTGACCT‐3′; GAPDH Reverse: 5′‐CCTGCTTCACCACCTTCTTG‐3′.

### 2.5 Western blotting

Cells were harvested and lysed in RIPA buffer containing protease inhibitors (Roche). Protein concentration was determined using the BCA protein assay kit (Pierce). Equal amounts of protein were separated by SDS‐PAGE and transferred onto PVDF membranes (Millipore). Membranes were blocked with 5% non‐fat milk in TBST for 1 h and incubated overnight at 4°C with primary antibodies against SLC7A11 (Abcam, ab175185), Transferrin (Cell Signaling Technology, #35293) and GAPDH (Cell Signaling Technology, #2118). Following incubation with HRP‐conjugated secondary antibodies (Cell Signaling Technology), bands were visualized using ECL detection reagents (Thermo Fisher Scientific).

### 2.6 Immunofluorescence (IF)

Cells grown on glass coverslips were fixed with 4% paraformaldehyde and permeabilized with 0.1% Triton X‐100. After blocking with 5% BSA, cells were incubated with anti‐SLC7A11 antibody overnight at 4°C. Subsequently, they were stained with Alexa Fluor 488‐conjugated secondary antibody (Thermo Fisher Scientific) and DAPI (Sigma) for nuclear staining. Images were captured using a fluorescence microscope (Olympus IX71).

### 2.7 CCK8 assay

Cell viability was assessed using the Cell Counting Kit‐8 (CCK8) assay (Dojindo Molecular Technologies). Following transfection, cells were seeded into 96‐well plates (1 × 104 cells/well) and incubated for 24, 48, or 72 h. CCK8 solution was added to each well, and absorbance was measured at 450 nm using a microplate reader (BioTek).

### 2.8 Colony formation assay

For colony formation, transfected cells were plated at a density of 500 cells per 6‐well plate and cultured for 10–14 days until visible colonies appeared. Colonies were fixed with methanol, stained with crystal violet (0.5% in methanol), and counted using an inverted microscope. The number of colonies formed was recorded and expressed as a percentage of the control group.

### 2.9 EdU assay

To evaluate cell proliferation, an EdU assay was performed using the EdU Cell Proliferation Kit (Thermo Fisher Scientific). Following transfection, cells were incubated with EdU for 2 h before fixation. Cells were then processed according to the manufacturer's instructions to visualize EdU incorporation. Images were obtained using a fluorescence microscope, and the percentage of EdU‐positive cells was calculated from at least three random fields.

### 2.10 Wound healing assay

Cells were seeded in six‐well plates and allowed to reach confluence. A sterile pipette tip was used to create a wound, and cells were washed with PBS. Fresh medium was added, and images were captured at 0‐ and 24‐h post‐wounding using an inverted microscope. The percentage of wound closure was calculated based on the areas measured.

### 2.11 Transwell invasion assay

For invasion assays, Matrigel‐coated Transwell inserts (Corning) were used. Cells were starved overnight and then resuspended in serum‐free RPMI‐1640. Approximately 1 × 10^5^ cells were placed in the upper chamber, and complete medium was added to the lower chamber. After 24 h, non‐invading cells were removed, and invading cells on the lower membrane were fixed, stained with crystal violet, and counted using an inverted microscope.

### 2.12 Xenograft tumor model

Male BALB/c nude mice (4–6 weeks old) were purchased from the Shanghai Laboratory Animal Center and housed in specific pathogen‐free conditions. All animal experiments were approved by the institutional animal care and use committee. For tumorigenesis studies, NCI‐H2122 cells (1 × 10^6^) transfected with either si‐NC or si‐SLC7A11 were subcutaneously injected into the right flank of each mouse. For the BEVs treatment, mice were divided into groups and weekly injected with PBS, BEVs‐LCTP, BEVs‐siSLC7A11, or BEVs‐LCTP‐siSLC7A11 at a dosage of 10 mg/kg via tail vein injection over a 21‐day treatment period. Tumor growth was monitored every 5 days, and volumes were calculated using the formula: Volume = (Length × Width^2^)/2.

### 2.13 Lung metastasis model

For metastasis evaluation, NCI‐H2122 cells were injected via the tail vein into BALB/c nude mice. For the BEVs treatment, mice were divided into groups and weekly injected with PBS, BEVs‐LCTP, BEVs‐siSLC7A11, or BEVs‐LCTP‐siSLC7A11 at a dosage of 10 mg/kg via tail vein injection over a 21‐day treatment period. After 4 weeks, the mice were euthanized, and lungs were collected for analysis. Pulmonary metastases were assessed through imaging, followed by histological examination.

### 2.14 Generation of BEVs

The BEVs were produced from genetically modified bacteria (Nissle1917) capable of expressing ClyA‐LCTP, SLC7A11 shRNA, and ClyA‐LCTP‐SLC7A11 shRNA. Bacteria were cultured in LB broth until they reached an appropriate optical density (OD600 of 0.6), then induced to express the desired constructs. The supernatant was harvested, and BEVs were purified via ultrafiltration and gradient centrifugation as previously described.^34^

### 2.15 Transmission electron microscopy (TEM)

BEVs were diluted in PBS and visualized by TEM. Samples were prepared by placing a drop on a copper grid, allowing it to adhere, then negatively staining with uranyl acetate. Imaging was performed using a JEOL JEM‐1400 Plus electron microscope.

### 2.16 Nanoparticle tracking analysis (NTA)

The size distribution and concentration of BEVs were quantified using NTA (NanoSight NS300). Samples were diluted appropriately and analyzed under controlled settings, with three independent measurements taken for each sample.

### 2.17 Real‐time PCR for SLC7A11 shRNA detection

To confirm the successful loading of SLC7A11 shRNA into BEVs, total RNA was extracted from the vesicles using TRIzol reagent. cDNA synthesis and real‐time PCR were conducted as previously described, using specific primers for SLC7A11 shRNA.

### 2.18 Histological analysis

Mice were divided into groups and weekly injected with PBS, BEVs‐LCTP, BEVs‐siSLC7A11, or BEVs‐LCTP‐siSLC7A11 at a dosage of 10 mg/kg via tail vein injection over a 21‐day treatment period. At predetermined time points, major organs (heart, liver, brain, kidneys, and lung) were harvested and fixed in 4% paraformaldehyde. Sections were prepared and stained with hematoxylin and eosin (H&E) for histopathological examination.

### 2.19 Biochemical analysis

Blood samples were collected via cardiac puncture, and serum was separated by centrifugation at 4000 rpm for 10 min. The serum was analyzed for biochemical markers of heart injury, liver and kidney function, including cardiac troponin T (cTnT), Creatine kinase‐MB (CK‐MB), alanine aminotransferase (ALT), aspartate aminotransferase (AST), creatinine, and blood urea nitrogen (BUN), and cytokines (IL‐1*β* and TNF‐*α*) using commercially available enzyme‐linked immunosorbent assay (ELISA) kits, according to the manufacturer's protocol.

### 2.20 Statistical analysis

Data were analyzed using GraphPad Prism software (version 9.0). All experiments were conducted in triplicate, and results are presented as the mean ± standard deviation (SD). Comparisons between groups were performed using Student's *t*‐test for two groups or one‐way ANOVA followed by Tukey's post hoc test for multiple comparisons. A *p*‐value of less than 0.05 was considered statistically significant.

## 3 RESULTS

### 3.1 Up‐regulated SLC7A11 expression in NSCLC tissues

**FIGURE 1:**
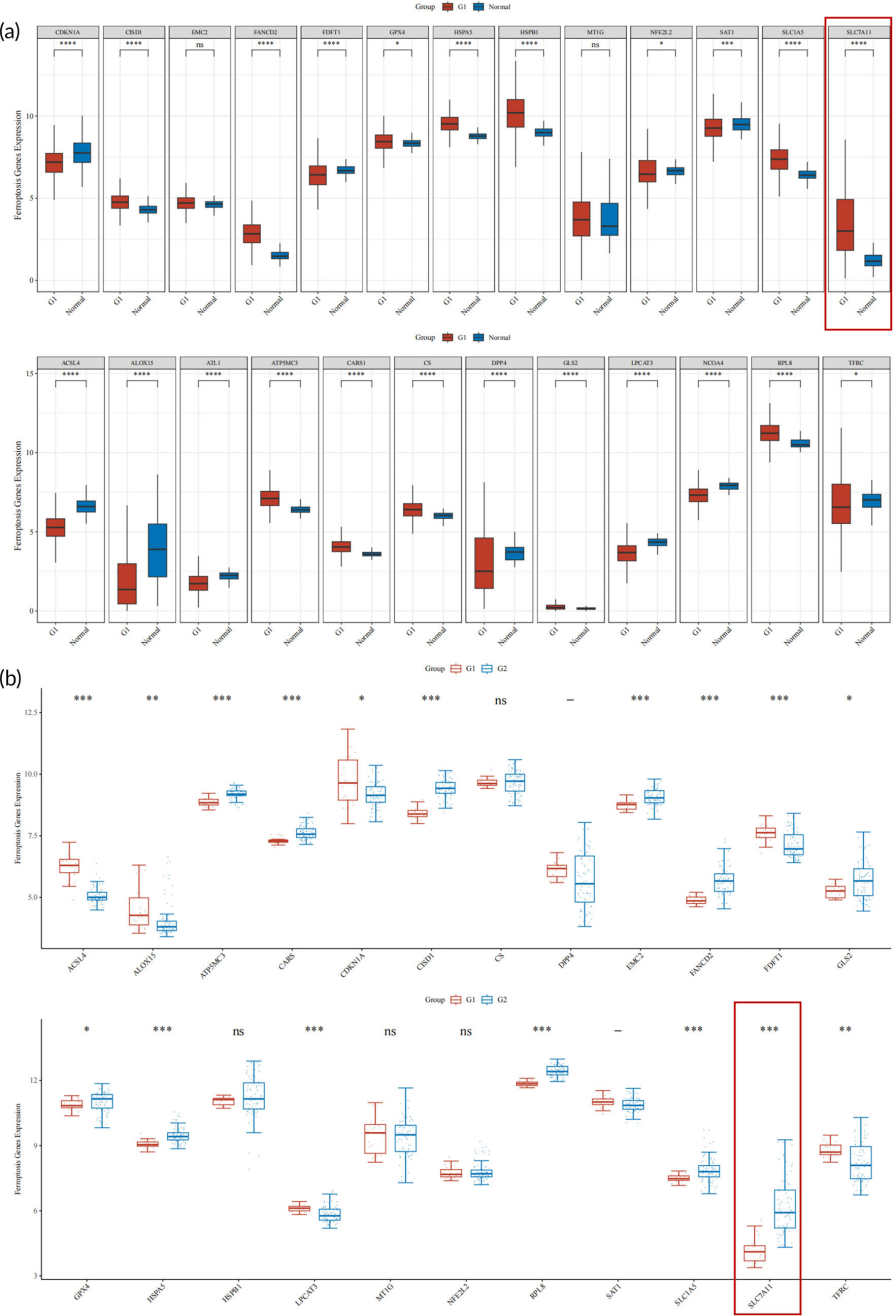
SLC7A11 was significantly up‐regulated in NSCLC tissues. (a) The expression levels of ferroptosis‐related genes in NSCLC were analyzed in the TCGA database. (B) The expression levels of ferroptosis‐related genes in NSCLC were analyzed in the GEO database (GSE33532).

To investigate the role of ferroptosis‐related genes in NSCLC, we analyzed expression levels using TCGA and GEO databases. Our analysis revealed that SLC7A11, a key regulator of cystine uptake and important for glutathione synthesis, was significantly up‐regulated in NSCLC tissues compared to normal lung tissues (Figure 1). In TCGA data, the expression of SLC7A11 was markedly higher in NSCLC samples (Figure 1a *p*< 0.01), and this finding was corroborated by GEO dataset analysis (GSE33532, Figure 1b), further establishing SLC7A11 as a potential therapeutic target in NSCLC.

### 3.2 Verification of SLC7A11 knockdown efficiency in NSCLC cells

**FIGURE 2:**
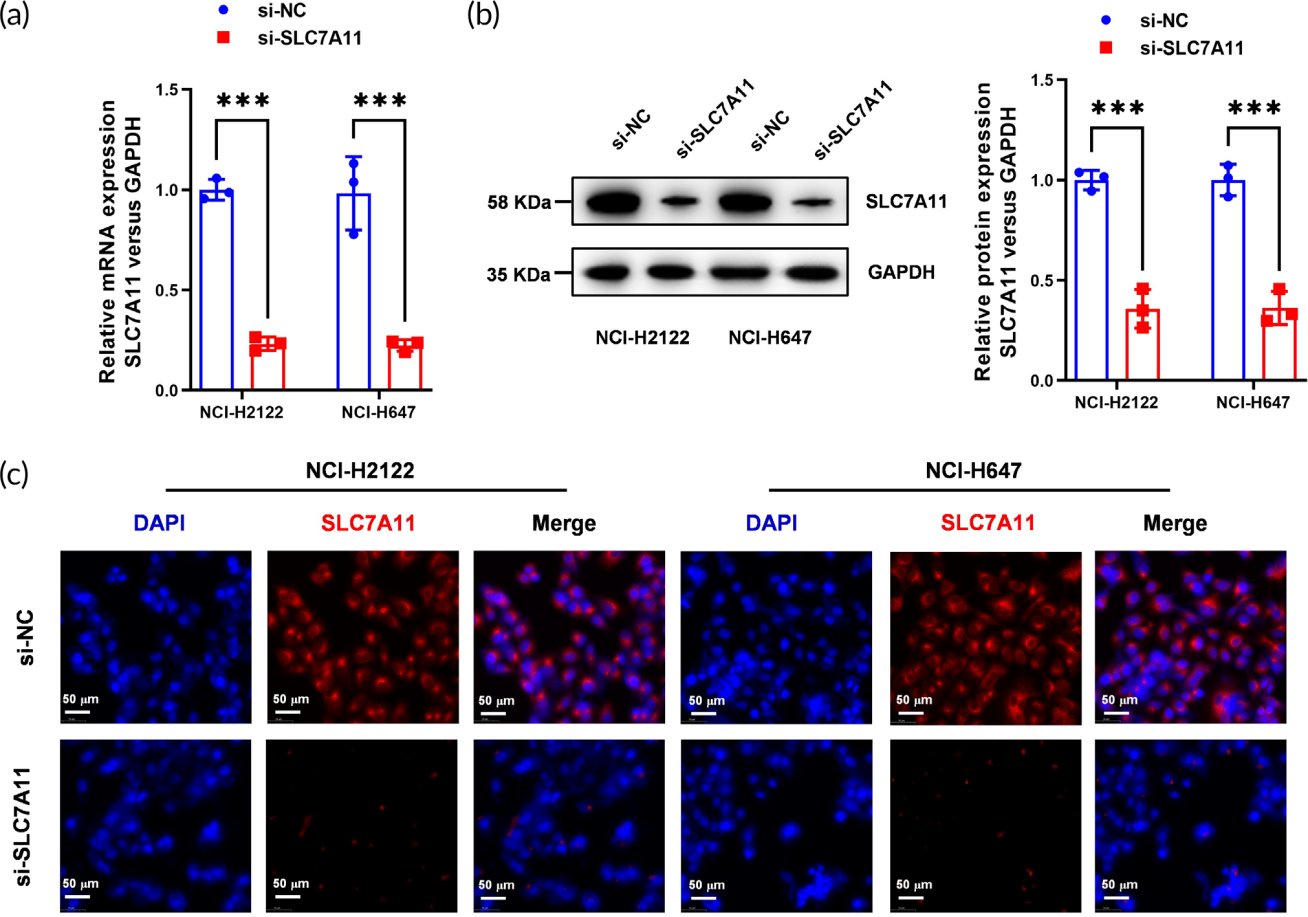
SLC7A11 knockdown efficiency in NSCLC cells was verified by qPCR, Western Blot, and IF. (a) Relative PCR was used to verify the knockdown efficiency of SLC7A11 in NSCLC cells (NCI‐H2122 and NCI‐H647). *N* = 3, ***p < 0.001. (b) Western blot was used to verify the knockdown efficiency of SLC7A11 in NSCLC cells. *N* = 3, ***p < 0.001. (c) IF was used to verify the knockdown efficiency of SLC7A11 in NSCLC cells. *N* = 3, Scale bar: 50 μm.

Subsequently, we evaluated the knockdown efficiency of SLC7A11 in two NSCLC cell lines, NCI‐H2122 and NCI‐H647, utilizing quantitative PCR (qPCR), western blotting, and IF techniques. The qPCR results demonstrated a significant reduction in SLC7A11 mRNA expression post‐siRNA treatment, with reductions of approximately 70% in both cell lines (Figure 2a). Western blot analysis supported these findings, showing a corresponding decrease in SLC7A11 protein levels (Figure 2b). Additionally, IF staining confirmed the diminished SLC7A11 localization in treated cells, reinforcing the effectiveness of our siRNA‐mediated knockdown strategy (Figure 2c).

### 3.3 Effects of SLC7A11 knockdown on NSCLC cell biology function

**FIGURE 3:**
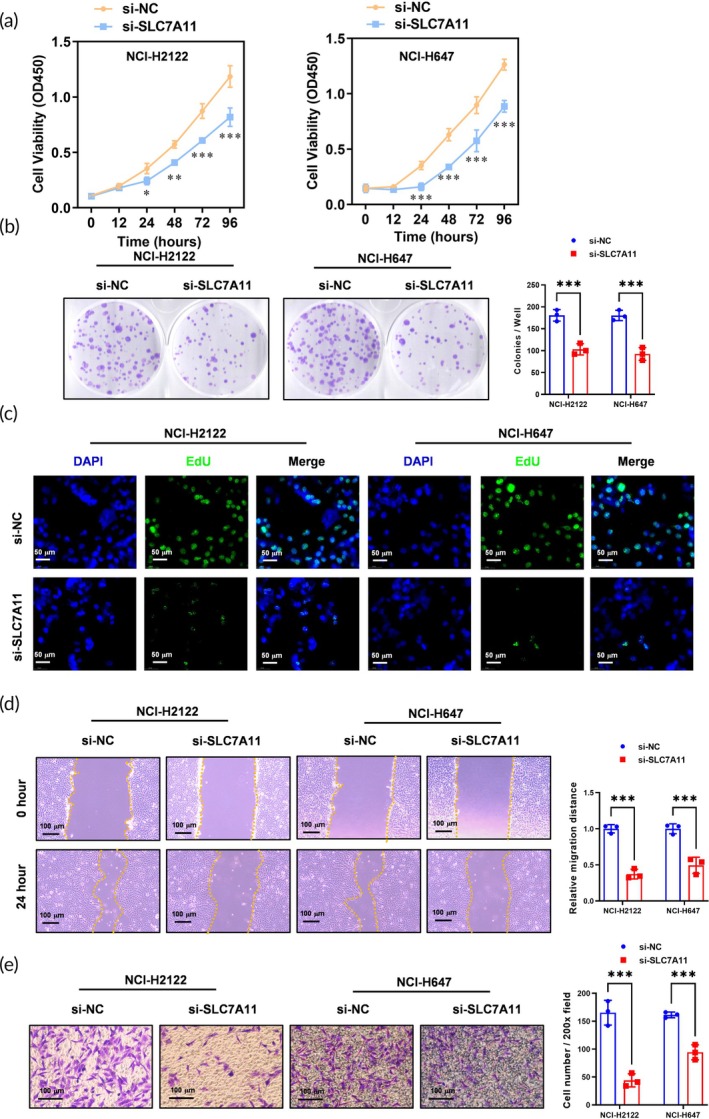
SLC7A11 knockdown impaired the proliferation, migration, and invasion abilities in NSCLC cell lines. (a) CCK8 assay was used to analyze the effect of SLC7A11 knockdown on the cell proliferation ability in NCI‐H2122 and NCI‐H647 cells. *N* = 3, *p < 0.05, **p < 0.01, ***p < 0.001. (b) Colony formation assay was used to verify the effect of SLC7A11 knockdown on the cell proliferation ability in NCI‐H2122 and NCI‐H647 cells. *N* = 3, ***p < 0.001. (c) EdU assay was used to verify the effect of SLC7A11 knockdown on the cell proliferation ability in NCI‐H2122 and NCI‐H647 cells. *N* = 3, Scale bar: 50 μm. (d) The effect of SLC7A11 knockdown on the migration ability of NCI‐H2122 and NCI‐H647 cells was evaluated using a wound healing assay. *N* = 3, Scale bar: 100 μm, ***p < 0.001. (e) The role of SLC7A11 knockdown on the invasion abilities of NCI‐H2122 and NCI‐H647 cells was analyzed by Transwell invasion assay. *N* = 3, Scale bar: 100 μm, ***p < 0.001.

Our next objective was to assess how SLC7A11 knockdown affected NSCLC cell behavior, focusing on proliferation, migration, and invasion capabilities. CCK‐8 assays indicated that SLC7A11 knockdown led to significantly reduced proliferation in both NCI‐H2122 and NCI‐H647 cells (Figure 3a), with a notable decrease in cell viability observed over 72 h. Consistent with these findings, colony formation assays revealed that si‐SLC7A11‐transfected cells formed fewer colonies compared to control groups (Figure 3b). EdU incorporation assays also showed a marked reduction in proliferative activity, confirming the impairment of cellular proliferation due to SLC7A11 silencing (Figure 3c).

In terms of cell migration and invasion, wound healing assays demonstrated that SLC7A11 knockdown significantly inhibited the migratory capacity of NSCLC cells, with healed areas being substantially larger in si‐NC treated controls than in si‐SLC7A11 groups (Figure 3d). Furthermore, Transwell invasion assays illustrated a similar trend, where the number of invading cells was drastically reduced upon SLC7A11 knockdown (Figure 3e). These results collectively indicate that silencing SLC7A11 disrupts critical tumorigenic behaviors in NSCLC cells.

### 3.4 Tumorigenesis impairment by SLC7A11 siRNA in xenograft models

**FIGURE 4:**
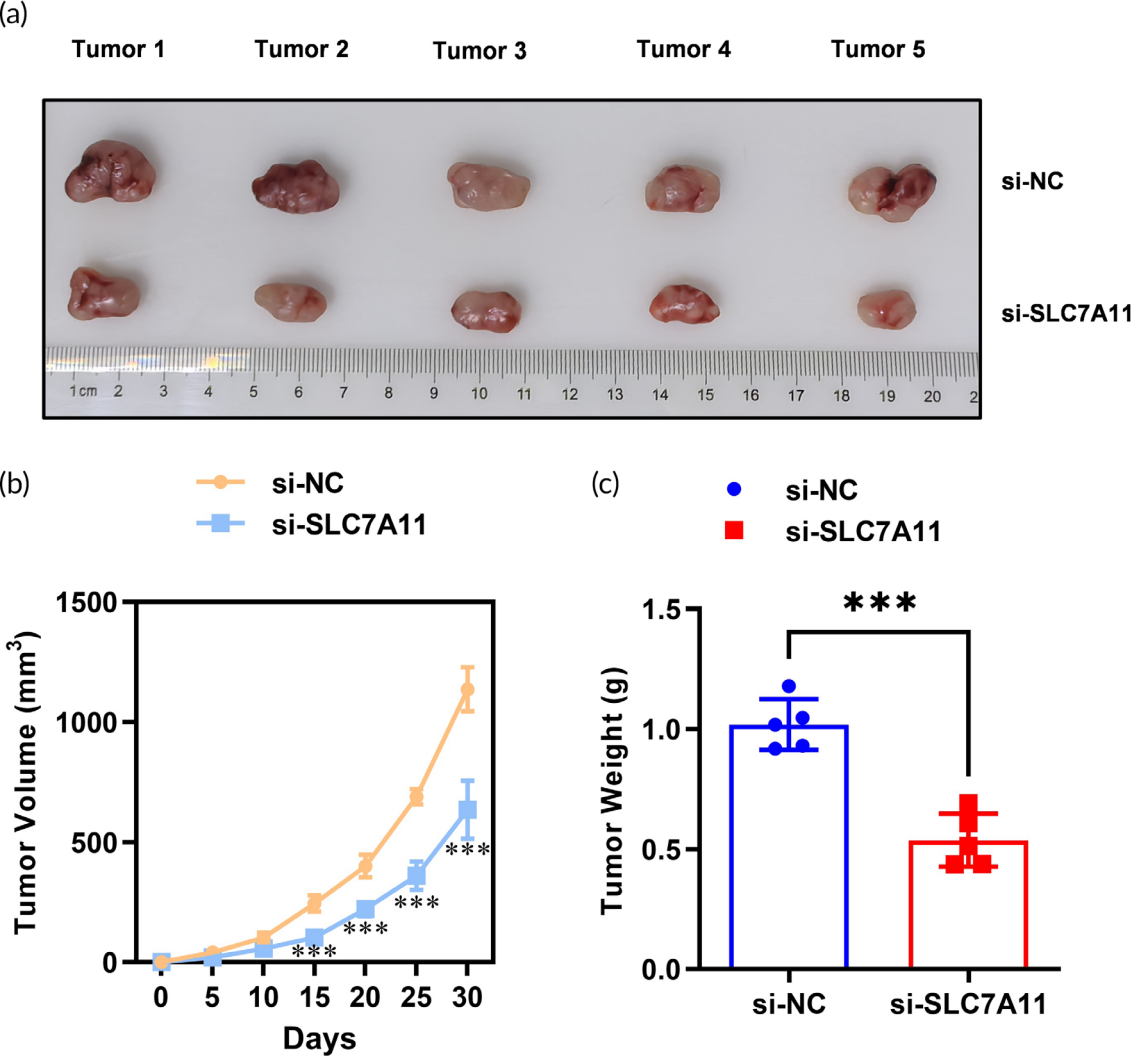
SLC7A11 knockdown impaired the tumorigenesis ability of NSCLC cells in the xenograft model. (a) A subcutaneous transplantation tumor model was used to evaluate SLC7A11 knockdown on the tumorigenesis of NSCLC cells. The generated tumor from NCI‐H2122 (si‐NC and si‐SLC7A11) cells was isolated and pictured. *N* = 5. (b) A growth curve of tumors generated from NCI‐H2122 cells was plotted every 5 days after subcutaneous injection. *N* = 5, ***p < 0.001. (c) The weight of tumors generated from NCI‐H2122 cells was analyzed. *N* = 5, ***p < 0.001.

We proceeded to assess the tumorigenic potential of NSCLC cells in vivo by employing subcutaneous xenograft models. Notably, tumors derived from NCI‐H2122 cells transfected with si‐SLC7A11 exhibited significantly reduced tumor volume and weight compared to control (si‐NC) groups (Figure 4a–c). The growth curve plotted every 5 days revealed a stark contrast between the two groups, emphasizing the impact of SLC7A11 knockdown on tumor development. Histological examination of excised tumors further confirmed that silencing SLC7A11 resulted in reduced cellular density within the tumors, which translated into smaller tumor sizes.

### 3.5 SLC7A11 siRNA impairs the metastatic potential in xenograft models

**FIGURE 5:**
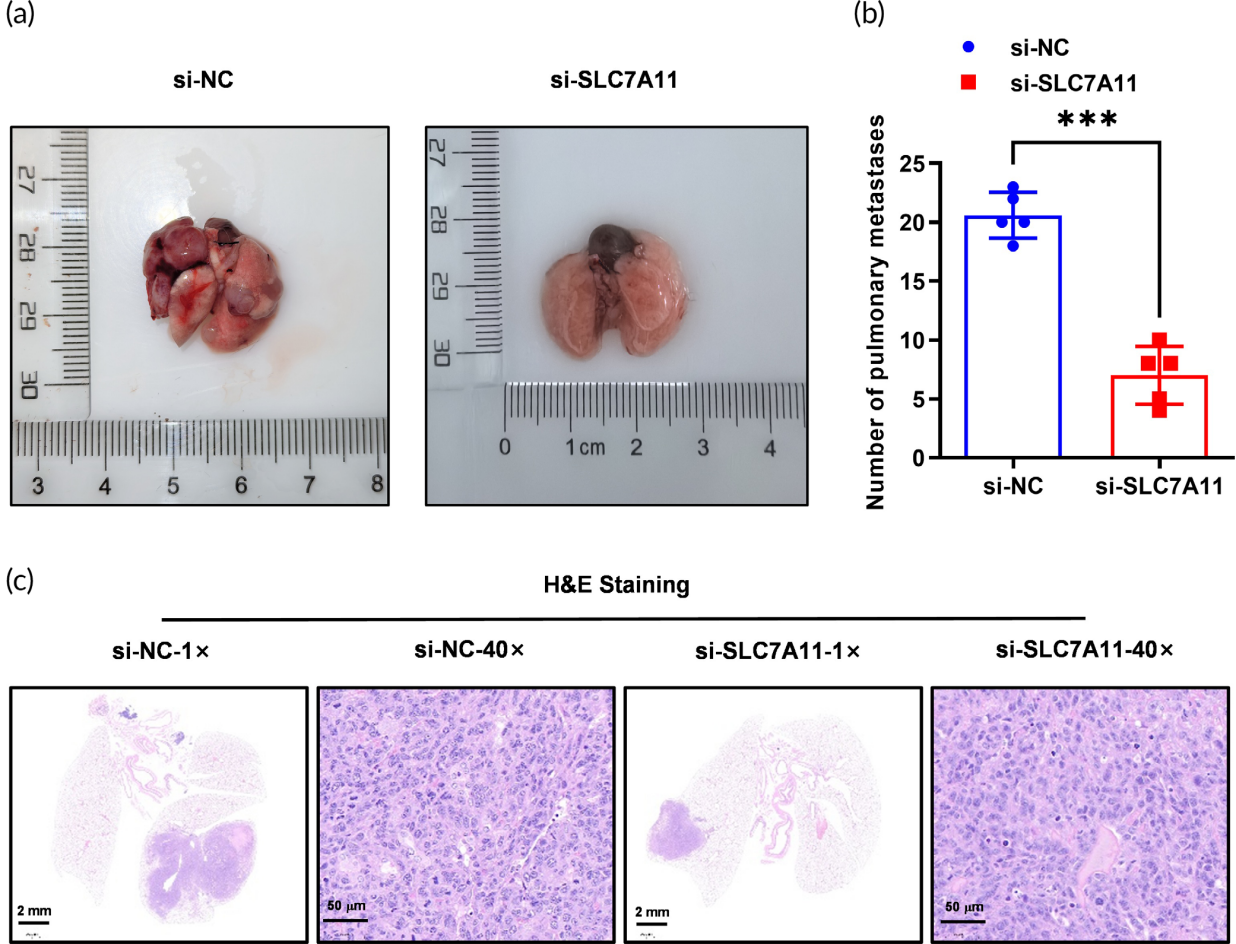
SLC7A11 knockdown impaired the tumor metastasis ability of NSCLC cells in the xenograft model. (a) A subcutaneous lung metastasis tumor model was used to evaluate SLC7A11 knockdown on the metastasis of NCI‐H2122 cells. The generated pulmonary metastases from NCI‐H2122 (si‐NC and si‐SLC7A11) cells were isolated and pictured. *N* = 5. (b) The number of pulmonary metastases was shown. *N* = 3, ***p < 0.001. (c) HE staining showed that SLC7A11 knockdown significantly reduced the number of pulmonary metastases. *N* = 5, Scale bar: 2 mm and 50 μm.

To evaluate the metastatic ability of NSCLC cells influenced by SLC7A11 knockdown, we established subcutaneous lung metastasis models. The results demonstrated that SLC7A11 knockdown led to a significant reduction in the number of pulmonary metastases generated from NCI‐H2122 cells (Figure 5a, b). H&E staining of lung tissues revealed a considerable decrease in metastatic foci in the si‐SLC7A11 group compared to controls (Figure 5c), indicating that targeting SLC7A11 effectively diminishes the metastatic spread of NSCLC.

### 3.6 Characterization and in vivo toxicity assay of engineered BEVs

**FIGURE 6:**
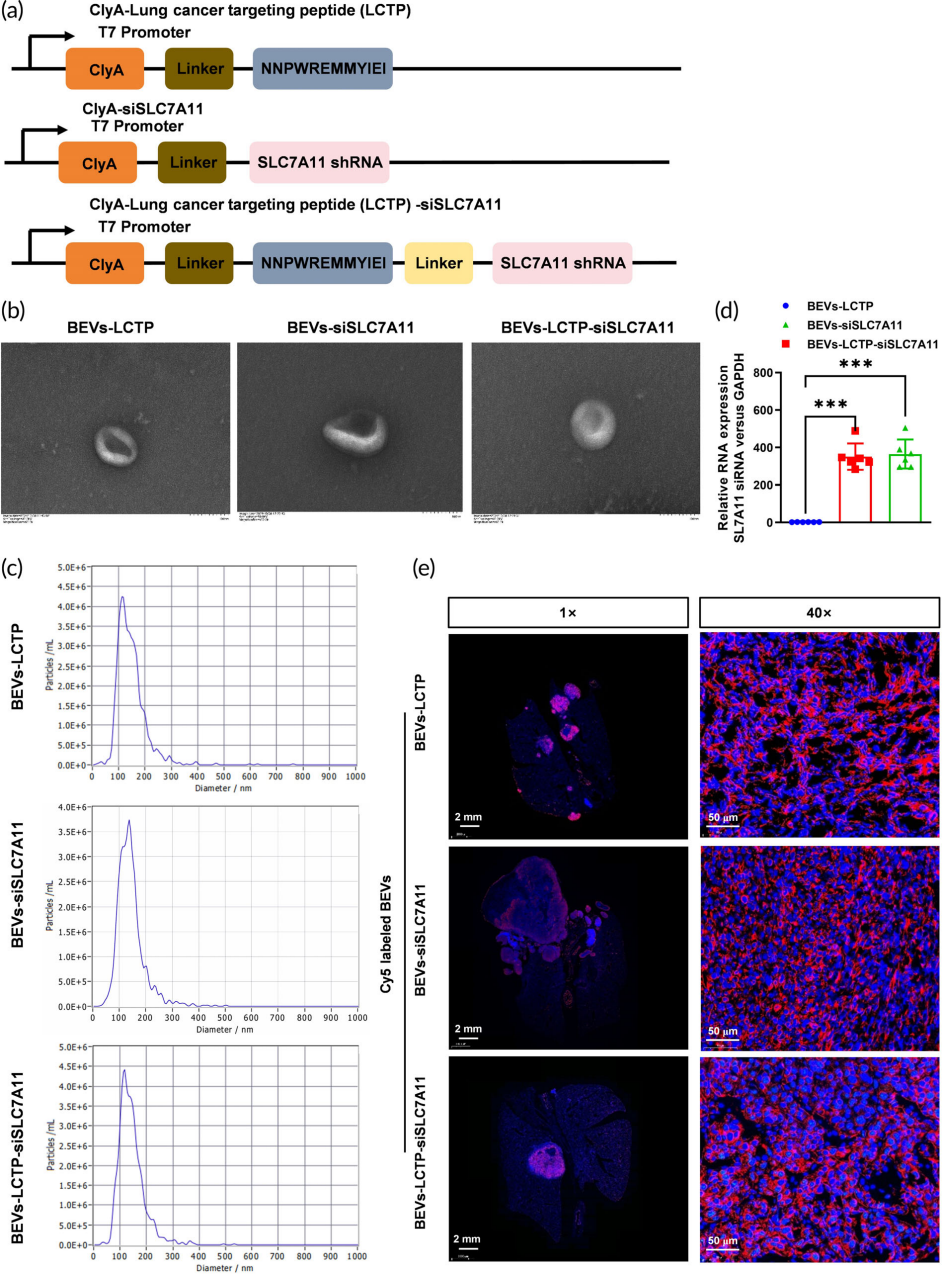
Design and characterization of engineered lung cell targeting and SLC7A11 siRNA‐expressing bacterial extracellular vesicles. (a) Schematic illustration of the construct used to express ClyA‐LCTP (NNPWREMMYIEI), ClyA‐SLC7A11‐shRNA (without targeting peptide) and ClyA‐LCTP (NNPWREMMYIEI)‐SLC7A11 shRNA. (b) TEM images of BEVs‐LCTP, BEVs‐siSLC7A11, and BEVs‐LCTP‐siSLC7A11. Scale bars represent 100 μm. *N* = 6. (c) NTA results of BEVs‐LCTP, BEVs‐siSLC7A11, and BEVs‐LCTP‐siSLC7A11. *N* = 6. (d) The expression level of SLC7A11 siRNA in the BEVs‐LCTP, BEVs‐siSLC7A11, and BEVs‐LCTP‐siSLC7A11 was verified by real‐time PCR. *N* = 6, ***p < 0.001. (e) Representative fluorescence microscopic images of the lung at 4 h after injection of Cy5‐labeled BEVs‐LCTP, BEVs‐siSLC7A11, and BEVs‐LCTP‐siSLC7A11. *N* = 6, Scale bar: 2 mm and 50 μm.

To improve targeted delivery of SLC7A11 siRNA, we engineered BEVs expressing SLC7A11‐specific shRNA. The design included a construct that facilitates lung targeting via ClyA‐LCTP fusion protein. The BEVs containing SLC7A11‐specific shRNA without LCTP fusion were used as a BEV controls (BEVs‐siSLC7A11). TEM confirmed the successful assembly and morphology of BEVs‐LCTP, BEVs‐siSLC7A11, and BEVs‐LCTP‐siSLC7A11 (Figure 6a,b). Nanoparticle tracking analysis (NTA) quantified the size distribution (BEVs‐LCTP median size: 139.5 ± 12.3 nm; BEVs‐siSLC7A11 median size: 131.3 ± 16.5 nm; BEVs‐LCTP‐siSLC7A11 median size: 134.6 ± 12.3 nm) and concentration (BEVs‐LCTP concentration: 2.2 × 10^11^ particles/mL; BEVs‐siSLC7A11 concentration: 1.9 × 10^11^ particles/mL; BEVs‐LCTP‐siSLC7A11 concentration: 2.6 × 10^11^ particles/mL), confirming the expected characteristics of the engineered vesicles (Figure 6c). Further validation through real‐time PCR demonstrated efficient loading and release of siRNA from the BEVs, confirming their suitability for RNA delivery (Figure 6d). The lung cancer targeting efficiency was analyzed by visualizing the Cy5 labeled BEVs (BEVs‐LCTP; BEVs‐siSLC7A11; BEVs‐LCTP‐siSLC7A11) in the subcutaneous lung metastasis models in vivo. Enriched Cy5 red signals were shown in the lung cancer metastasis tumors (Figure 6e).

**FIGURE 7:**
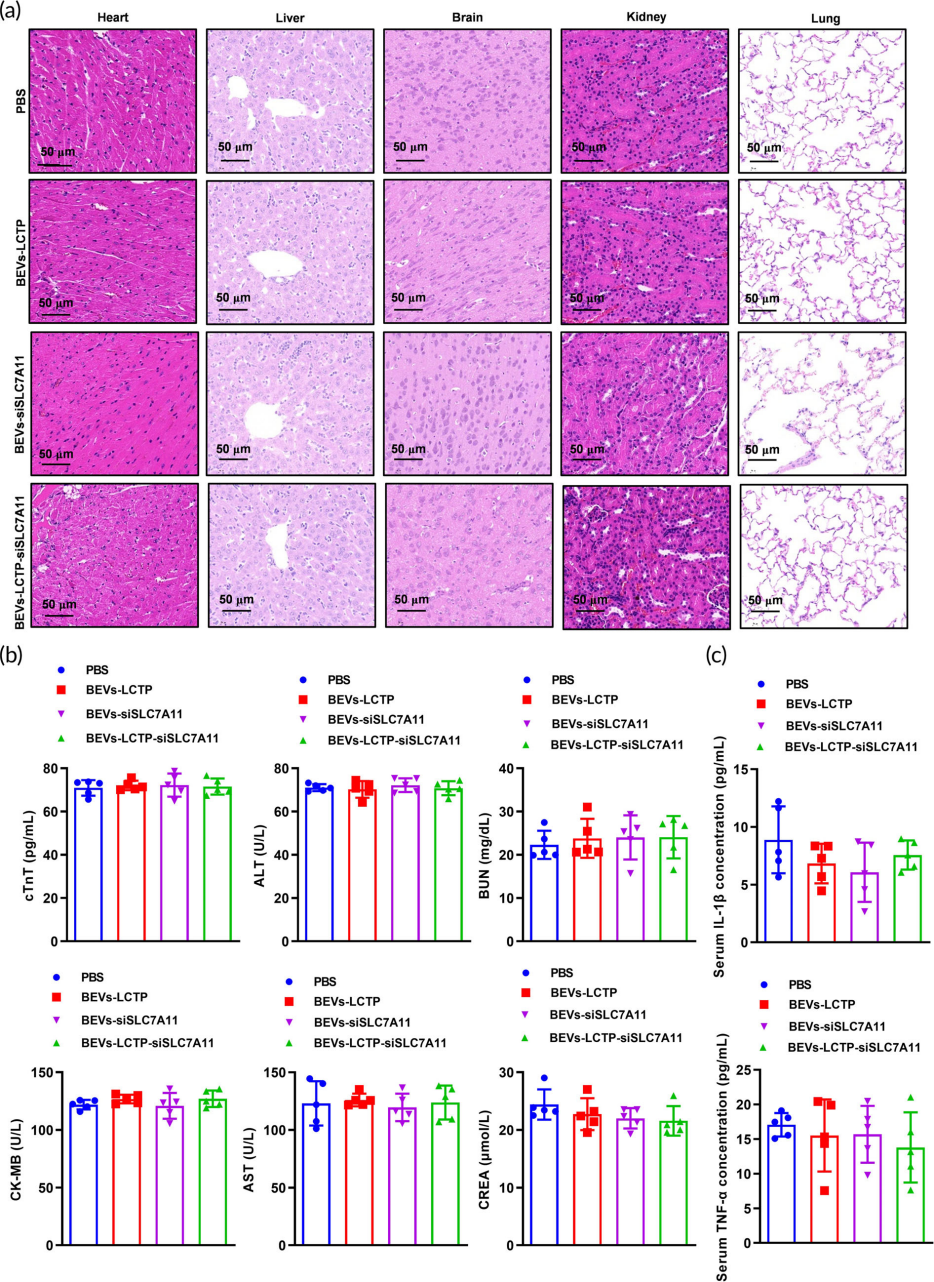
In vivo toxicity assay of bioengineered BEVs. (a) H&E staining of the heart, liver, brain, kidney, and lung after administration of PBS, BEVs‐LCTP, BEVs‐siSLC7A11, and BEVs‐LCTP‐siSLC7A11. *N* = 5, Scale bar: 50 μm. (b) Toxicity test of cardiac troponin T (cTnT), Creatine kinase‐MB (CK‐MB), alanine transaminase (ALT), aspartate aminotransferase (AST), blood urea nitrogen (BUN), and creatinine (CREA). *N* = 5. (C) The serum cytokines (IL‐1*β*, TNF‐*α*) in treated mice were analyzed by ELISA assays. *N* = 5.

To evaluate the systemic effects and potential toxicity of our treatment approach, we monitored body weight changes and hematological parameters throughout the study. No significant differences in body weight were observed between treatment groups, suggesting that our engineered BEVs (BEVs‐LCTP; BEVs‐siSLC7A11; BEVs‐LCTP‐siSLC7A11) did not induce substantial adverse effects (Figure 7a). Serum biochemical assays indicated no significant alterations in liver and kidney function markers, indicating good biocompatibility of the BEVs during the treatment regimen (Figure 7b). Furthermore, ELISA assays indicated no significant alterations in the serum cytokines (IL‐1*β*, TNF‐*α*) in treated mice, suggesting minimal immune activation by the BEVs (Figure 7c).

### 3.7 BEVs‐LCTP‐siSLC7A11 impaired the tumorigenesis and metastasis abilities a of NSCLC cells and activated the ferroptosis in the xenograft model

**FIGURE 8:**
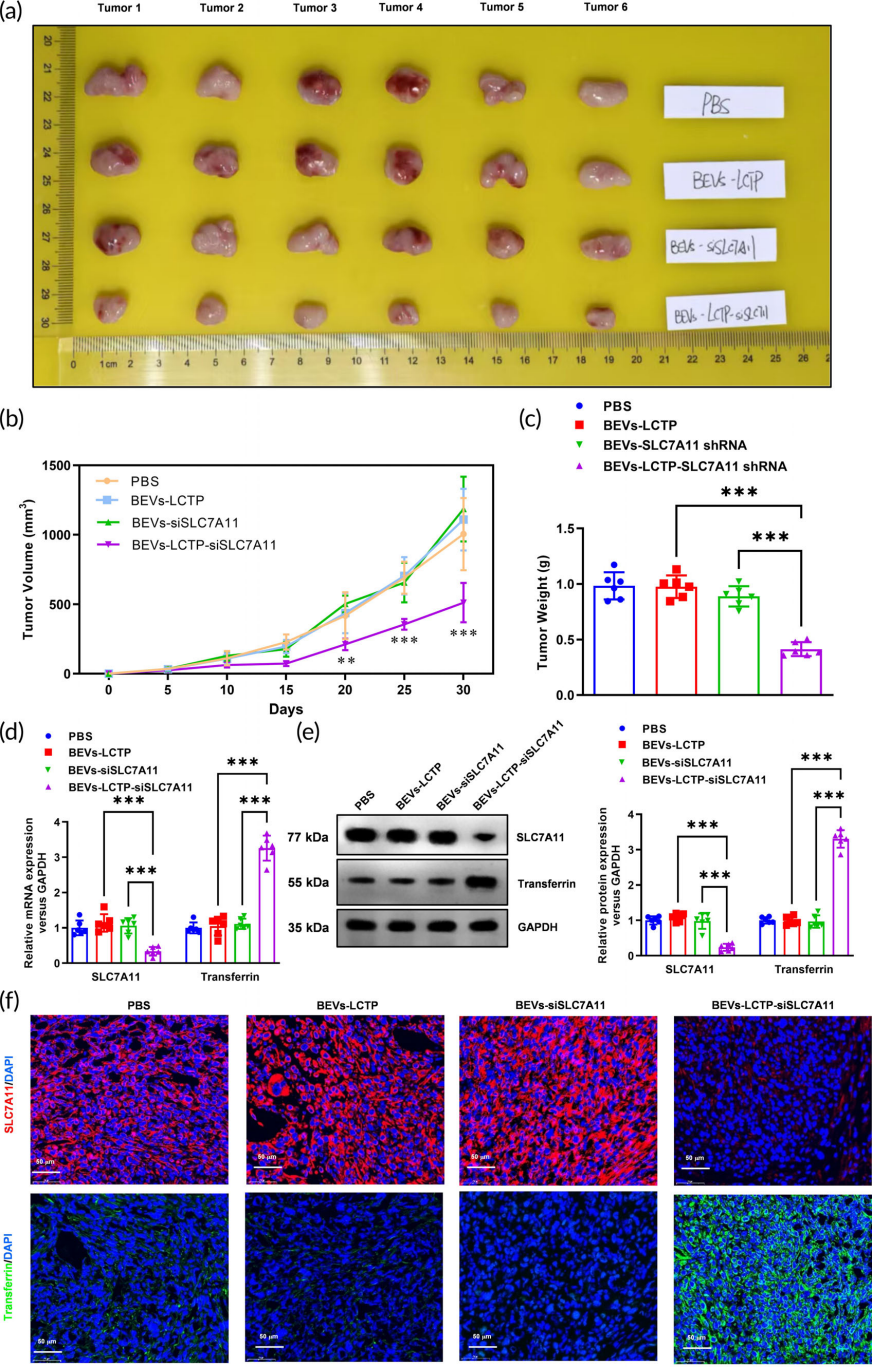
BEVs‐LCTP‐siSLC7A11 impaired the tumorigenesis ability of NSCLC cells and activated the ferroptosis in the xenograft model. (a) The subcutaneous transplantation tumor model was used to evaluate PBS, BEVs‐LCTP, BEVs‐siSLC7A11, and BEVs‐LCTP‐siSLC7A11 on the tumorigenesis of NSCLC cells. The generated tumor from NCI‐H2122 cells was isolated and pictured. *N* = 6. (b) A growth curve of tumors generated from NCI‐H2122 cells was plotted every 5 days after subcutaneous injection. *N* = 6, ***p < 0.001. (c) The weight of tumors generated from NCI‐H2122 cells was analyzed. *N* = 6, ***p < 0.001. (d) Real‐time qPCR was used to confirm the down‐regulation of the SLC7A11 and ferroptosis marker transferrin mRNA levels. *N* = 6, ***p < 0.001. (e) Western blotting was used to confirm the down‐regulation of the SLC7A11 and ferroptosis marker transferrin protein levels. *N* = 6, ***p < 0.001. (f) The expression of SLC7A11 and ferroptosis marker transferrin in the tumors was analyzed by Immunofluorescence. *N* = 6, Scale bar: 50 μm.

**FIGURE 9:**
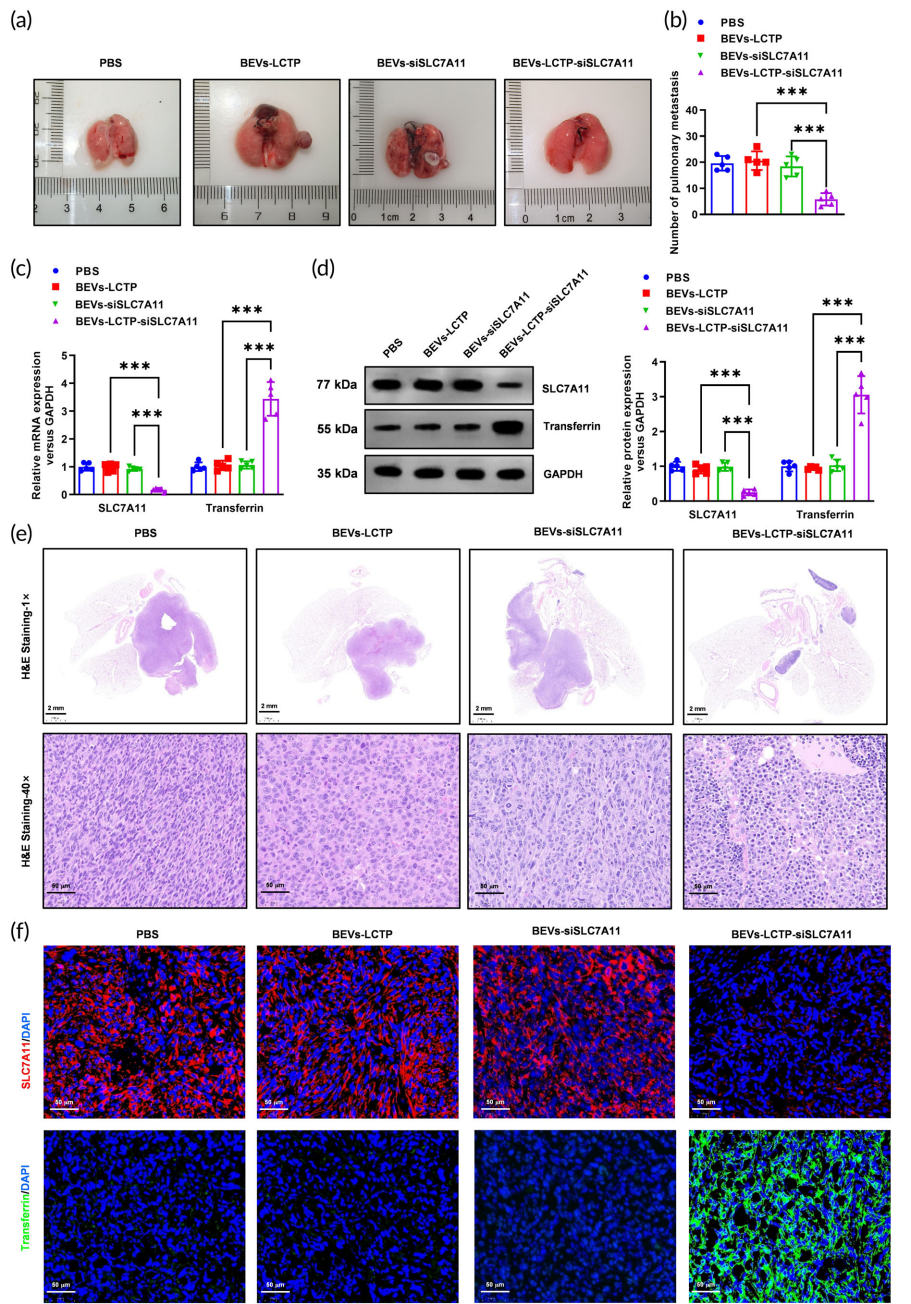
BEVs‐LCTP‐siSLC7A11 impaired the tumor metastasis ability of NSCLC cells and activated ferroptosis in the xenograft model. (a) A subcutaneous lung metastasis tumor model was used to evaluate PBS, BEVs‐LCTP, BEVs‐siSLC7A11, and BEVs‐LCTP‐siSLC7A11 on the metastasis of NCI‐H2122 cells. The generated pulmonary metastases from NCI‐H2122 cells were isolated and pictured. *N* = 5. (b) The number of pulmonary metastases was shown. *N* = 5, ***p < 0.001. (c) Real‐time qPCR was used to confirm the down‐regulation of the SLC7A11 and ferroptosis marker transferrin mRNA levels. *N* = 5, ***p < 0.001. (d) Western blotting was used to confirm the down‐regulation of the SLC7A11 and ferroptosis marker transferrin protein levels. *N* = 5, ***p < 0.001. (e) HE staining showed that SLC7A11 knockdown significantly reduced the number of pulmonary metastases. *N* = 5, Scale bar: 2 mm and 50 μm. (f) The expression of SLC7A11 and ferroptosis marker transferrin in the metastases was analyzed by Immunofluorescence. *N* = 5, Scale bar: 50 μm.

To evaluate the therapeutic potential of SLC7A11‐targeting nanoparticles, NCI‐H2122 xenograft mice were weekly administered BEVs‐siSLC7A11 and BEVs‐LCTP‐siSLC7A11 at graded doses (0.3, 1, 3, 10, 30 mg/kg) over a 21‐day treatment period. Quantitative real‐time PCR analysis revealed distinct dose–response profiles between the two formulations. While BEVs‐siSLC7A11 showed no significant suppression of SLC7A11 mRNA levels at doses below 30 mg/kg, a marked reduction (*p* < 0.05) was observed at the highest dose of 30 mg/kg in lung metastasis tumors (Supplementary Figure 1A). In contrast, BEVs‐LCTP‐siSLC7A11 demonstrated dose‐dependent inhibition of SLC7A11 mRNA expression across the entire tested range (1–30 mg/kg), achieving statistical significance at all dose levels (Supplementary Figure 1A). These transcriptional changes were corroborated at the protein level through Western blot analysis, which showed corresponding decreases in SLC7A11 protein expression (Supplementary Figure 1B). Based on these pharmacodynamic findings, a submaximal dose of 10 mg/kg was selected for subsequent functional studies to balance efficacy with potential toxicity concerns. To investigate the therapeutic efficacy of BEVs‐LCTP‐siSLC7A11, dual xenograft models were established. In subcutaneous tumors (Figure 8), BEVs‐LCTP‐siSLC7A11 treatment markedly inhibited tumorigenesis, reducing tumor volume by 49% (***p < 0.001 vs. PBS, Figure 8b) and tumor weight by 58% (***p < 0.001, Figure 8c) compared to controls. Strikingly, this suppression coincided with molecular evidence of ferroptosis activation: qPCR and Western blot revealed concurrent downregulation of SLC7A11 and upregulation of transferrin, a ferroptosis marker, at both mRNA (***p < 0.001, Figure 8d) and protein levels (Figure 8e). IF further confirmed diminished SLC7A11/transferrin co‐expression in treated tumors (Figure 8f). In the metastatic model (Figure 9a), BEVs‐LCTP‐siSLC7A11 exhibited potent anti‐metastatic activity, reducing pulmonary nodule counts by 61% (***p < 0.001, Figure 9b) and inducing histological regression of metastases (HE staining, Figure 9e). Mechanistically, metastatic lesions showed parallel reductions in SLC7A11 and increased transferrin expression via qPCR (***p < 0.001, Figure 9c), Western blot (Figure 9d), and IF (Figure 9f), consistent with systemic ferroptosis activation. Notably, BEVs‐LCTP‐siSLC7A11 outperformed both naked BEVs‐LCTP and non‐targeted BEVs‐siSLC7A11 across all endpoints, highlighting the critical role of ligand‐conjugated delivery in achieving therapeutic efficacy.

## 4 DISCUSSION

The findings of this study elucidate the potential therapeutic implications of targeting the SLC7A11 gene in NSCLC using engineered BEVs. The data presented here indicate that SLC7A11 is significantly upregulated in NSCLC tissues, and its knockdown impairs various cancer hallmarks, including proliferation, migration, invasion, tumorigenesis, and metastasis. Moreover, the use of BEVs expressing SLC7A11 siRNA presents a novel strategy for targeted gene silencing in lung cancer therapy, promoting ferroptosis as a mechanism to inhibit tumor progression.

Our analysis of publicly available datasets from TCGA and GEO revealed that SLC7A11 expression was markedly elevated in NSCLC tissues compared to normal lung tissues. This finding aligns with previous studies suggesting that SLC7A11 plays a critical role in cancer biology by regulating the cellular uptake of cysteine, which is essential for glutathione synthesis and resistance to oxidative stress.^35^, ^36^, ^37^ The upregulation of SLC7A11 in NSCLC suggests an adaptive response of these tumors to maintain redox balance and promote survival under oxidative conditions.^19^, ^38^ In the context of cancer, high levels of SLC7A11 have been associated with increased resistance to therapies, as well as enhanced metastatic capabilities.^39^ Targeting SLC7A11 could therefore represent a viable approach to sensitize NSCLC cells to conventional therapies and address the problem of treatment resistance.

To explore the functional consequences of SLC7A11 inhibition, we employed diverse techniques including qPCR, Western blotting, and IF to confirm successful knockdown of SLC7A11 in NSCLC cell lines NCI‐H2122 and NCI‐H647. Each technique corroborated the efficiency of our siRNA‐mediated knockdown approach. Notably, this multi‐faceted validation underscores the robustness of our experimental design and provides confidence in the authenticity of subsequent findings regarding the biological implications of SLC7A11 inhibition.

In examining the effects of SLC7A11 knockdown on cell behavior, we observed significant impairments in proliferation, migration, and invasion capabilities of NSCLC cells. The CCK8 assay demonstrated reduced cell viability, while colony formation assays confirmed diminished proliferative capacity upon SLC7A11 knockdown. Additionally, EdU incorporation assays illustrated a substantial decrease in DNA synthesis, indicating that SLC7A11 contributes not only to proliferation but also to maintaining the overall growth signal within NSCLC cells. Furthermore, wound healing assays and Transwell invasion assays revealed that SLC7A11 knockdown substantially inhibited the migratory and invasive properties of NSCLC cells. These findings suggest that SLC7A11 is integral to the epithelial‐to‐mesenchymal transition, a critical process underlying cancer metastasis. It is plausible that SLC7A11 enhances cellular plasticity, allowing cancer cells to adaptively respond to microenvironmental cues that promote metastatic spread.

To further investigate the translational relevance of SLC7A11 knockdown, we utilized xenograft models to assess its impact on tumorigenesis. Subcutaneous transplantation of NCI‐H2122 cells with SLC7A11 knockdown led to significantly reduced tumor growth and weight compared to control groups. The generation of these tumor models provided crucial insight into the role of SLC7A11 in vivo, demonstrating its contribution to tumor growth dynamics. Importantly, these results highlight the feasibility of targeting SLC7A11 as a therapeutic strategy to impair tumor development in NSCLC. Metastasis remains one of the leading causes of mortality in NSCLC patients. Our investigations into the effects of SLC7A11 knockdown on metastatic potential reveal profound insights. Using a lung metastasis model, we showed that the reduction of SLC7A11 not only decreased the number of pulmonary metastases but also affected the histological characteristics of the metastases themselves. HE staining further illustrated that SLC7A11 knockdown resulted in a marked reduction in the metastatic burden in the lungs.

These findings are particularly relevant as they underscore the dual role of SLC7A11 in both primary tumor growth and metastatic spread. By diminishing the migratory and invasive capacities of NSCLC cells, SLC7A11 knockdown could serve as a promising strategy to combat metastasis, which often complicates treatment outcomes in lung cancer patients. One of the standout innovations of this study is the engineering of BEVs for targeted delivery of SLC7A11 siRNA. A critical innovation of this study lies in the strategic selection of BEVs as siRNA delivery vehicles, a choice predicated on their distinct advantages over mammalian‐derived extracellular vehicles (EVs) and synthetic nanoparticle systems.^28^ While human cell‐derived EVs have demonstrated therapeutic potential, their clinical translation faces inherent limitations: mammalian EV production requires expensive cell culture systems with low yield (∼1–10 μg/mL), presents batch‐to‐batch variability, and carries risks of horizontal transfer of oncogenic cargo from cancer‐derived EVs.^31^ In contrast, BEVs address these challenges through (1) *Scalability*—Gram‐negative bacterial cultures enable high‐yield production (50–200 μg/mL) using cost‐effective fermentation techniques^29^; (2) *Safety*—BEVs lack endogenous mammalian surface markers (e.g., MHC complexes), minimizing immune clearance and off‐target interactions^30^; and (3) *Engineerability*—The modular structure of BEVs permits reliable functionalization, as demonstrated by our LCTP ligand conjugation (NNPWREMMYIEI)^40^ for tumor‐targeted delivery. The characterization of these BEVs demonstrates their potential as vehicles for targeted gene therapy.^34^, ^41^, ^42^ The unique properties of BEVs, including their biocompatibility, ability to traverse biological barriers, and inherent capacity for delivering nucleic acids, position them as a promising platform for cancer treatment.^32^, ^33^, ^43^ In our experiments, we assessed the efficacy of BEVs derived from *Escherichia coli* Nissle 1917^44^ loaded with SLC7A11 siRNA in NSCLC cells. Flow cytometry analysis confirmed successful uptake of BEVs by both NCI‐H2122 and NCI‐H647 cells, demonstrating the feasibility of using these engineered vesicles for intracellular delivery. Subsequent evaluation of SLC7A11 expression confirmed significant knockdown, validating the functionality of BEVs in mediating siRNA delivery. Moreover, functional assays indicated that BEV‐mediated delivery of SLC7A11 siRNA led to reductions in cell viability, proliferation, migration, and invasion similar to those observed with direct transfection of siRNA. This suggests that BEVs can effectively deliver therapeutic payloads and elicit a biological response comparable to established transfection methods while potentially offering advantages such as reduced toxicity and enhanced targeting specificity.

An intriguing aspect of our findings is the role of ferroptosis, a form of regulated cell death characterized by iron‐dependent lipid peroxidation, upon SLC7A11 knockdown.^25^, ^27^, ^38^, ^45^, ^46^ Our results indicate that inhibition of SLC7A11 leads to decreased cysteine availability, thereby diminishing glutathione synthesis and promoting oxidative stress within NSCLC cells. Consequently, this resulted in increased sensitivity to ferroptosis, further implicating SLC7A11 as a pivotal regulator of redox homeostasis in lung cancer. To investigate this phenomenon, we treated NSCLC cells post‐knockdown with ferroptosis inducers and inhibitors. The data revealed that cells with SLC7A11 knockdown exhibited heightened susceptibility to ferroptosis, suggesting that targeting SLC7A11 may enhance the effectiveness of ferroptosis agents and represent a viable strategy for sensitizing NSCLC cells to oxidative stress‐based therapies. The potential of targeting SLC7A11 in NSCLC through engineered BEVs presents exciting opportunities for clinical translation. Given the high prevalence of NSCLC and its associated mortality rates, novel therapeutic approaches are urgently needed. The ability to harness BEVs for targeted delivery not only overcomes some limitations associated with conventional siRNA therapies, such as off‐target effects and systemic toxicity but also provides a means to achieve localized treatment with enhanced efficacy. Future studies will be essential to optimize the engineering of BEVs for improved targeting capabilities, enhance the loading efficiency of therapeutic agents, and evaluate long‐term effects and biocompatibility in vivo. Furthermore, exploring the combination of BEV‐mediated SLC7A11 knockdown with existing chemotherapy or immunotherapy regimens could offer synergistic effects, improving patient outcomes.

**FIGURE 10:**
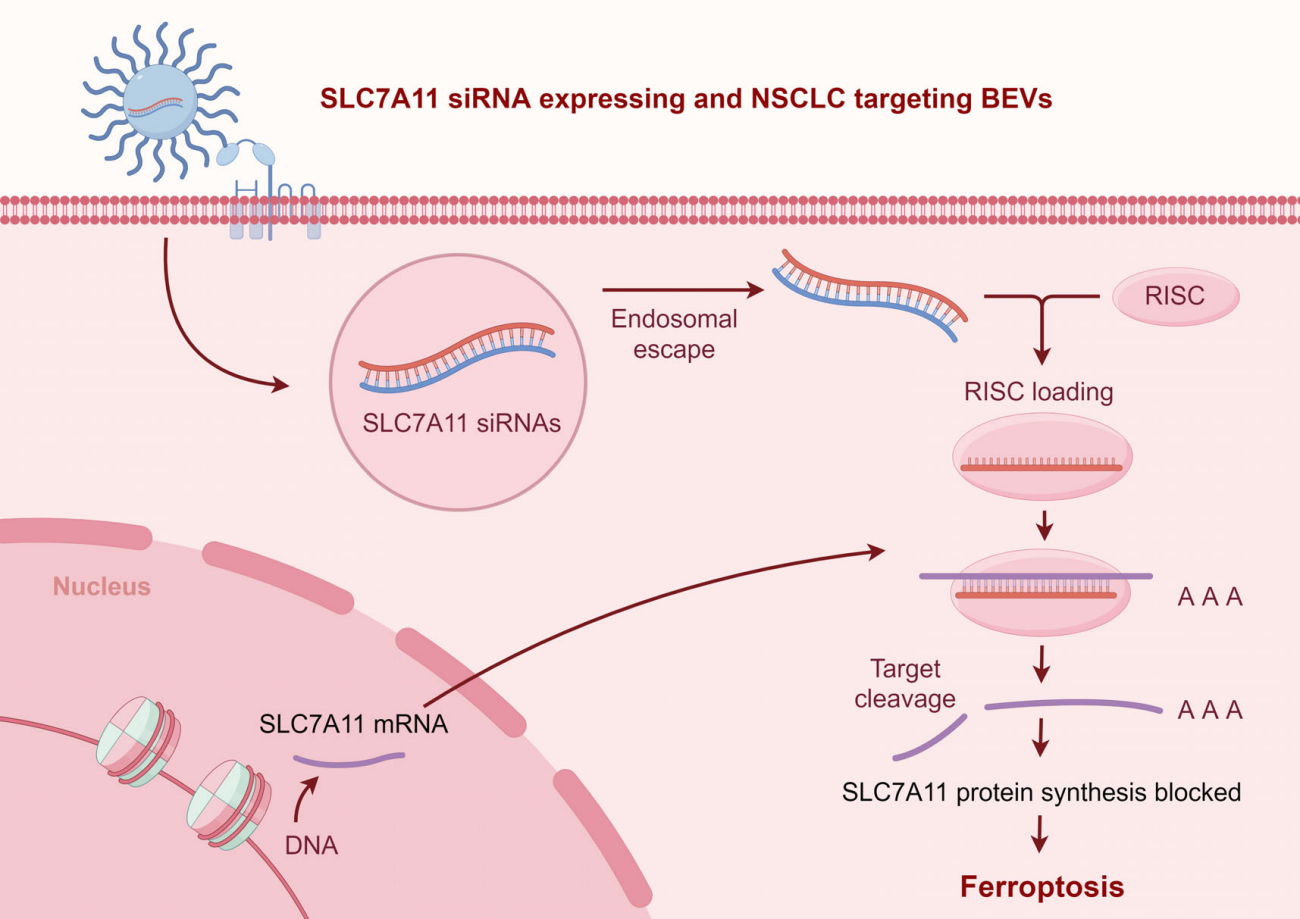
Schematic diagram of Design and characterization of Engineered lung cell targeting and SLC7A11 siRNA expressing bacterial extracellular vesicles and its therapeutic strategy for NSLCL via activating ferroptosis.

In summary, our study underscores the significance of SLC7A11 as a therapeutic target in non‐small cell lung cancer. The development of engineered BEVs as a means to deliver SLC7A11 siRNA provides a novel approach to combat tumor progression and metastasis. By elucidating the relationship between SLC7A11 expression and cancer hallmarks such as proliferation, migration, invasion, and ferroptosis, we propose that targeting this gene may serve as an impactful strategy in the fight against NSCLC (Figure 10). The integration of innovative delivery systems like BEVs into therapeutic strategies holds promise for advancing precision medicine and improving outcomes for patients with lung cancer. Further research in this domain will be vital to translate these findings from bench to bedside.

## AUTHOR CONTRIBUTIONS

X. Wang and H. Xu were primarily responsible for designing the experimental protocols, analyzing the data, and drafting the initial manuscript. X. Zhou played a pivotal role in conducting the experiments, interpreting the results, and providing critical feedback during manuscript preparation. J. Liu assisted in data collection and preliminary analysis. H. Xu supervised the entire project, including the conception of the research idea, coordination of team efforts, and finalization of the manuscript. They also ensured the integrity of the research and were instrumental in securing funding and resources for the study.

## FUNDING INFORMATION

This work was supported by grants from the National Natural Science Foundation of China (82260054).

## CONFLICT OF INTEREST STATEMENT

The authors declare no conflict of interest.

## Supporting information


**Supplementary Figure 1.** BEVs‐LCTP‐siSLC7A11 dose‐dependently reduced the tumor SLC7A11 levels. BEVs were administered at 10 mg/kg weekly for 3 weeks in the NCI‐H2122 xenograft mice. The tumor tissues were harvested and the (A) Quantitative real‐time PCR analysis and (B) Western Blotting were used the assess the levels of SLC7A11 mRNA and protein. *N* = 6, *p < 0.05, **p < 0.01, ***p < 0.001.

## Data Availability

The data that support the findings of this study are available from the corresponding author upon reasonable request.
