# Supplementary material for: Engineered lung cell targeting and SLC7A11 siRNA expressing bacterial extracellular vesicles impair the progression of none‐small cell lung cancer
Source: Bioeng Transl Med. 2025 Apr 16;10(5):e70021. doi: 10.1002/btm2.70021 (PMC12478443; doi:10.1002/btm2.70021)

**Supplementary Figure 1. BEVs-LCTP-siSLC7A11 dose-dependently reduced the tumor SLC7A11 levels.** BEVs were administered at 10 mg/kg weekly for 3 weeks in the NCI-H2122 xenograft mice. The tumor tissues were harvested and the (A) Quantitative real-time PCR analysis and (B) Western Blotting were used the assess the levels of SLC7A11 mRNA and protein. N=6, **P*<0.05, ***P*<0.01, ****P*<0.001.


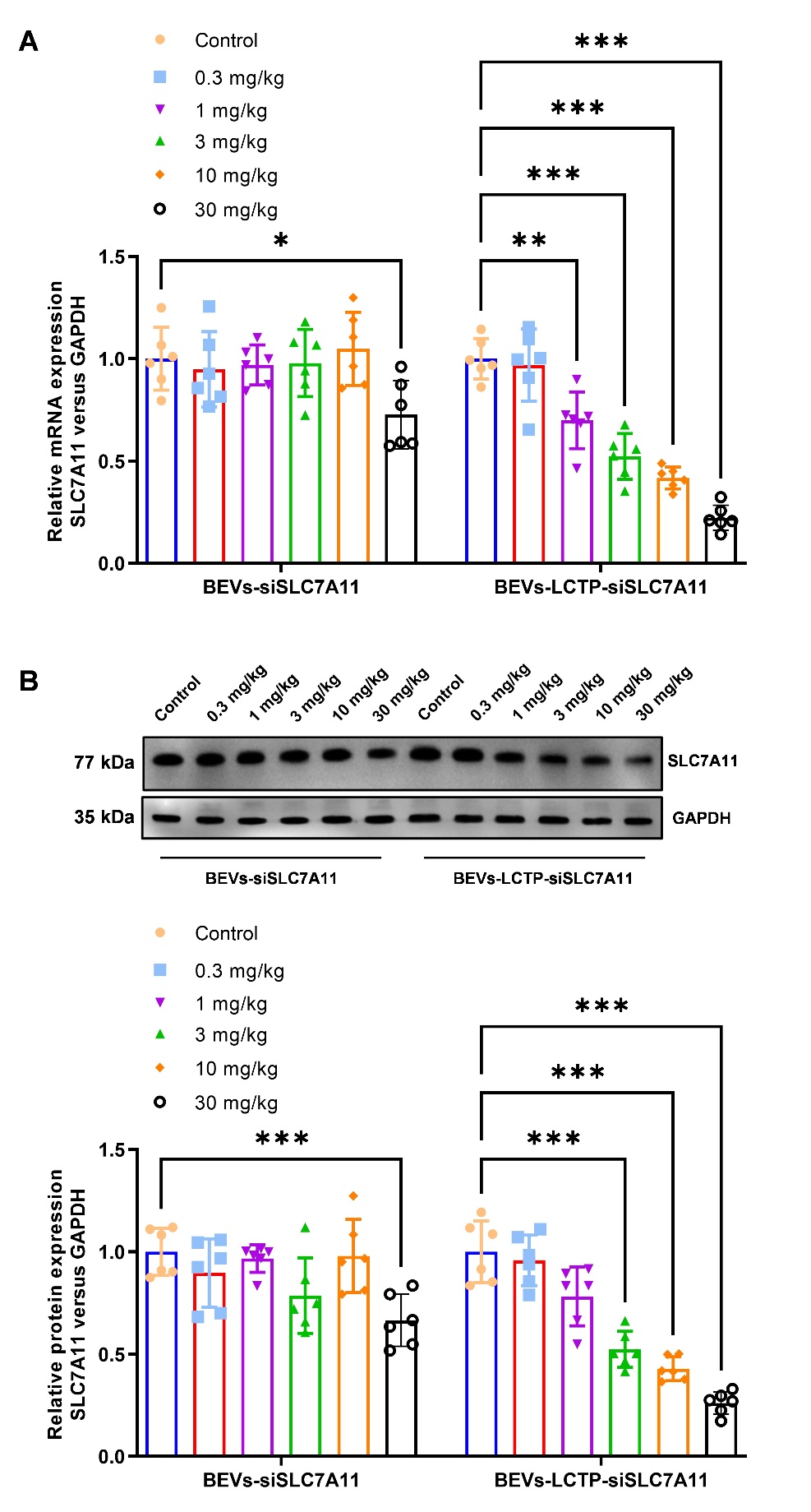

Supplement: Supplementary file 1 — Supplementary Figure 1. BEVs‐LCTP‐siSLC7A11 dose‐dependently reduced the tumor SLC7A11 levels. BEVs were administered at 10 mg/kg weekly for 3 weeks in the NCI‐H2122 xenograft mice. The tumor tissues were harvested and the (A) Quantitative real‐time PCR analysis and (B) Western Blotting were used the assess the levels of SLC7A11 mRNA and protein. N = 6, *p < 0.05, **p < 0.01, ***p < 0.001. [file BTM2-10-e70021-s001.docx]
